# Antenatal infection and intraventricular hemorrhage in preterm infants: A meta-analysis

**DOI:** 10.1097/MD.0000000000016665

**Published:** 2019-08-02

**Authors:** Jinglan Huang, Junjie Meng, Imti Choonara, Tao Xiong, Yibin Wang, Huiqing Wang, Yi Qu, Dezhi Mu

**Affiliations:** a Department of Pediatrics, West China Second University Hospital; b Key Laboratory of Birth Defects and Related Diseases of Women and Children of the Ministry of Education, Sichuan University, Chengdu, Sichuan, China; c Academic Division of Child Health, University of Nottingham, Derbyshire Children's Hospital, Derby, UK; d Deep Underground Space Medical Center, West China Hospital, Sichuan University, Chengdu, Sichuan, China.

**Keywords:** antenatal, infants, infection, IVH, preterm

## Abstract

**Background:**

The aim of this study was to summarize current evidence evaluating the association between antenatal infection and intraventricular hemorrhage (IVH) in preterm infants.

**Materials and methods:**

We searched for published articles on antenatal infection and IVH in 3 English (PubMed, the Cochrane Library, and EBSCO) and 3 Chinese (VEIPU, CNKI, and WANFANG) databases on May 19, 2019. In addition, the references of these articles were screened. The included studies had to meet all of the following criteria: preterm infants (<37 weeks); comparing antenatal infection with no infection; the outcomes included IVH (all grades), mild IVH, or sereve IVH; the type of study was randomized controlled trial or cohort study.

**Results:**

A total of 23 cohort studies involving 13,605 preterm infants met our inclusion criteria. Antenatal infection increased the risk of IVH (odds ratios ([OR] 2.18, 95% confidence intervals [CI] 1.58–2.99), mild IVH (OR 1.95, 95% CI 1.09–3.49) and severe IVH (OR 2.65, 95% CI 1.52–4.61). For type of antenatal infection, the ORs and 95% CI were as follows: 2.21 (1.60–3.05) for chorioamnionitis, 2.26 (1.55–3.28) for histologic chorioamnionitis, 1.88 (1.22–2.92) for clinical chorioamnionitis, and 1.88 (1.14–3.10) for ureaplasma.

**Conclusions:**

Antenatal infection may increase the risk of developing IVH in the preterm infant. The evidence base is however of low quality and well-designed studies are needed.

## 1 Introduction

Preterm birth is the leading cause of neonatal death and under-five mortality worldwide.^[1]^ Intraventricular hemorrhage (IVH), one of most common complication of preterm birth, is a major risk factor for death and neurodevelopmental disabilities in preterm infants.^[2]^ Despite the improvement of neonatal intensive care in the last few decades, the morbidity of IVH has not declined, mainly because of a significant increase in survival rates of premature neonates.^[3]^ The incidence of IVH ranges from 25% to 45% in preterm infants weighing <1500 g.^[3–5]^ Mortality rates were 4%, 10%, 18%, and 40%, respectively, for grades I, II, III, and IV IVH during initial hospitalization.^[6]^ Among survivors, both mild (grade I and II) and severe IVH (grade III and IV) are associated with high risk of moderate–severe neurodevelopmental impairment.^[2,3]^ Average hospital cost per infants has also increased from $201,578 to $353,554 in the past decade, which places a tremendous burden on affected families.^[6]^ Currently, the risk factors for IVH are not completely clear. Established risk factors include small gestational age (GA) and low birth weight (LBW).^[7]^

Antenatal infection has been reported to be an important risk factor for preterm delivery. It is responsible for 40% of premature deliveries.^[8]^ Recent research indicates that exposure to intrauterine infection/inflammation results in more serious injury than preterm delivery alone. It is associated with complications including neonatal sepsis,^[9]^ bronchopulmonary dysplasia, and patent ductus arteriosus.^[10,11]^

A relationship between antenatal infection and IVH has been widely supported by pathophysiological mechanism from scientific research. It involves interactions between strong immunological reactions and inflammatory cascades.^[12–14]^ Previous studies have suggested that inflammatory factors may ultimately lead to the occurrence of IVH through elevation of cerebral oxygen consumption,^[15–18]^ breakdown of the brain barriers,^[19]^ and activation of the immune response.^[20]^ Besides the effects of inflammatory factors, the unstable blood pressure of the brain by infection may also contribute to the development of IVH. Premature infants lack mature autoregulation function of cerebral blood pressure.^[21]^ Infection and sepsis may induce abnormal fluctuations of blood pressure, resulting in unstable cerebral blood pressure, leading to an increase in the risk of IVH.^[22]^ Recently, clinical studies have reported a relationship between antenatal infection and IVH.^[18,23]^ To date, there has been no systematic review regarding the relationship between antenatal infection and IVH. Thus, we systematically reviewed the current evidence evaluating the effects of antenatal infection on the risk of IVH in preterm infants.

## 2 Material and methods

### 2.1 Search strategy

We searched for published articles on antenatal infection and IVH in 3 English (PubMed, the Cochrane Library, and EBSCO) and three Chinese (VEIPU, CNKI, and WANFANG) databases on May 19, 2019. In addition, the references of the included studies were also screened. We used the keywords (“preterm” OR “premature”) AND (“chorioamnionitis” OR “infection” OR “inflammation” OR “amnionitis” OR “amnionitides” OR “funisitis” OR “funisitides” OR “sepsis” OR “pyemia” OR “pyohemia” OR “pyaemia” OR “septicemia” OR “poisoning, blood” OR “blood poisoning”) AND (“cerebral intraventricular hemorrhages” OR “hemorrhage, cerebral intraventricular” OR “intraventricular hemorrhage, cerebral” OR “cerebral intraventricular haemorrhage” OR “haemorrhage, cerebral intraventricular” OR “intraventricular haemorrhage, cerebral”) to search for and select studies including the target population.

### 2.2 Study selection

Two researchers (JLH and JJM) independently searched for and screened all the citations identified by the above searches by reviewing their titles and abstracts. Then, the full texts of the relevant studies were retrieved. The included studies had to meet all of the following criteria: preterm infants (<37 weeks); comparing antenatal infection with no infection; the outcomes included IVH (all grades), mild IVH, or severe IVH; the type of study was randomized controlled trial (RCT) or cohort study.

We excluded case–control studies, cross-section studies, case reports, commentary articles, editorials, and animal research.

### 2.3 Data extraction

Two investigators (JLH and JJM) performed separate data extractions using a structured data extraction sheet. The following data were extracted from each study: authors, year of publication, country, study design, GA, birth weight, IVH grade, infection type, and number of participants. Studies approved by both investigators were included in the meta-analysis.

### 2.4 Quality assessment

For RCTs, we would use the criteria outlined in the Cochrane Handbook for Systematic Reviews of interventions.^[24,25]^ However, no RCTs were identified. The quality of observational studies was assessed by the Newcastle-Ottawa Scale (NOS).^[26,27]^ NOS involves the 2 investigators rating the studies by scores for the quality of the studies’ study group selection, study group comparability, and ability to assess the outcome of interest.^[27]^ Studies were divided into high-quality (scores of 9) and low-quality (scores of 1–8).^[26]^ Any discrepancies regarding study quality were discussed and resolved by a third author.

### 2.5 GRADE assessment

A “Summary of finding” table was prepared to evaluate the quality of the evidence. Observational studies were graded as low-certainty evidence. The quality was downgraded if there were limitations, inconsistencies, indirectness, imprecision and other considerations, or upgraded to high and moderate if there was large effect or a dose–response gradient.

### 2.6 Statistical analysis

We conducted the statistical analyses with Cochrane Collaboration's Review manager 5.3 (Cochrane Collaboration, UK) and Stata 12.0 (StataCorp, College Station, Texas). The associations between antenatal infection and IVH were expressed as odds ratios (OR) with corresponding 95% confidence intervals (CI). Between-study statistical heterogeneity was assessed using the Q statistic (significant at *P* < .1) and *I*^2^ values (values of 25%, 50%, and 75% represented low, moderate, and high heterogeneity, respectively). The random-effects model was selected when the *I*^2^ ≥50% or *P* < .1, otherwise, the fixed-effects model was used.

We conducted sensitivity analysis by: removing low-quality studies; removing the baseline imbalance studies in GA (the *P* value < .05 or without *P* value among groups), and re-analyzing the remaining studies, to assess the stability of the results. And we evaluated the subgroups to explore the possible heterogeneity and the *I*^2^ and *P* value were used to represent subgroup difference. Then, we performed funnel plots and Egger test to assess publication bias in each of the pooled study groups when ≥5 included studies were available.^[28]^

## 3 Results

### 3.1 Study characteristics

**Figure 1:**
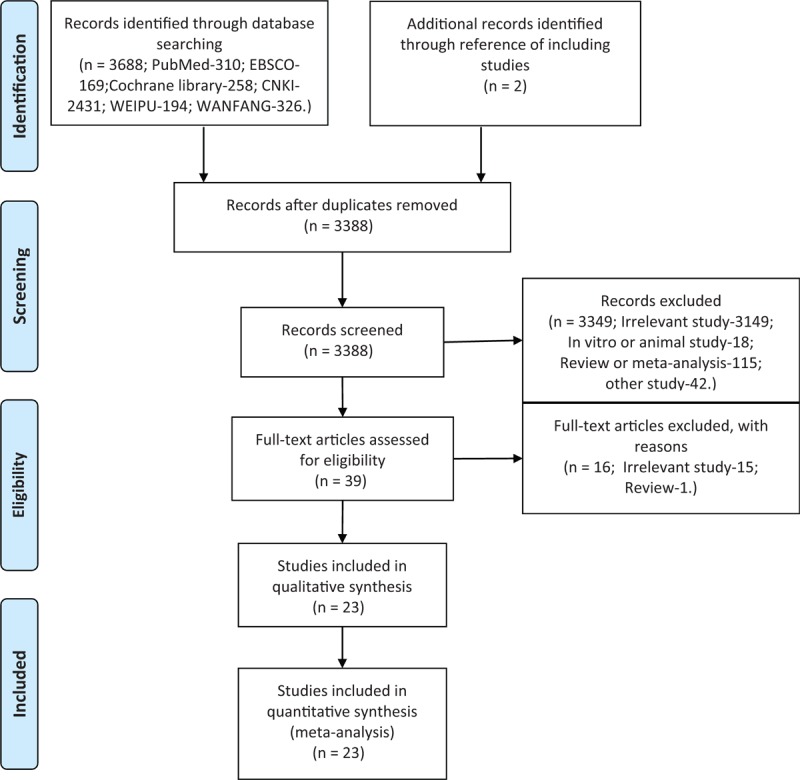
Flow diagram showing the results of search strategy.

We identified 3688 publications published between each database's date of inception and May 19, 2019. We excluded 302 duplicate studies. A total of 3349 of the above studies were excluded by title and abstract. We subjected the remaining 39 studies to a full-text review. Fifteen studies were excluded as there was no relevant comparison and 1 study was excluded as it was a review. Ultimately, we pooled data from 23 studies involving 13,605 preterm infants^[18,23,29–49]^ for the meta-analysis (Fig. 1).

The majority of studies were published after 2000. The sample size of included studies ranges from 62 to 5849. The average GA was under 33 weeks, and average birth weight was <1900 g in included studies.

In 23 studies reporting IVH as outcome for preterm infants, 10 studies reported mild IVH (grade I and II); 14 studies reported severe IVH (grade III and IV), 11 studies did not report information regarding the grades of IVH. Twenty-one studies reported data on chorioamnionitis. Two studies reported data on ureaplasma. The characteristics of the included studies are shown in Table 1.

**Table 1:**
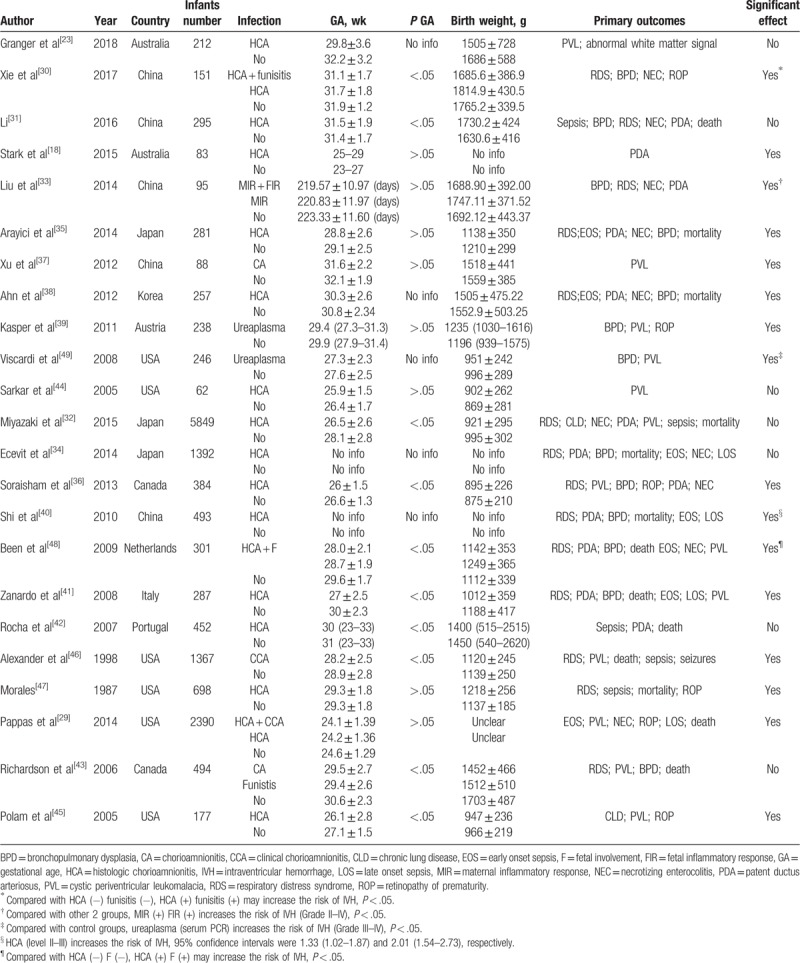
Characteristics of included studies.

### 3.2 Quality assessment

All the studies in the meta-analysis were cohort studies. Based on our assessment, 11 studies^[18,23,30,31,33,35,37–39,44,49]^ were rated as high-quality studies (scores of 9), and 12 studies^[28,32,34,36,40–43,45–48]^ were rated as low-quality studies (scores of 7–8) (Table 2).

**Table 2:**
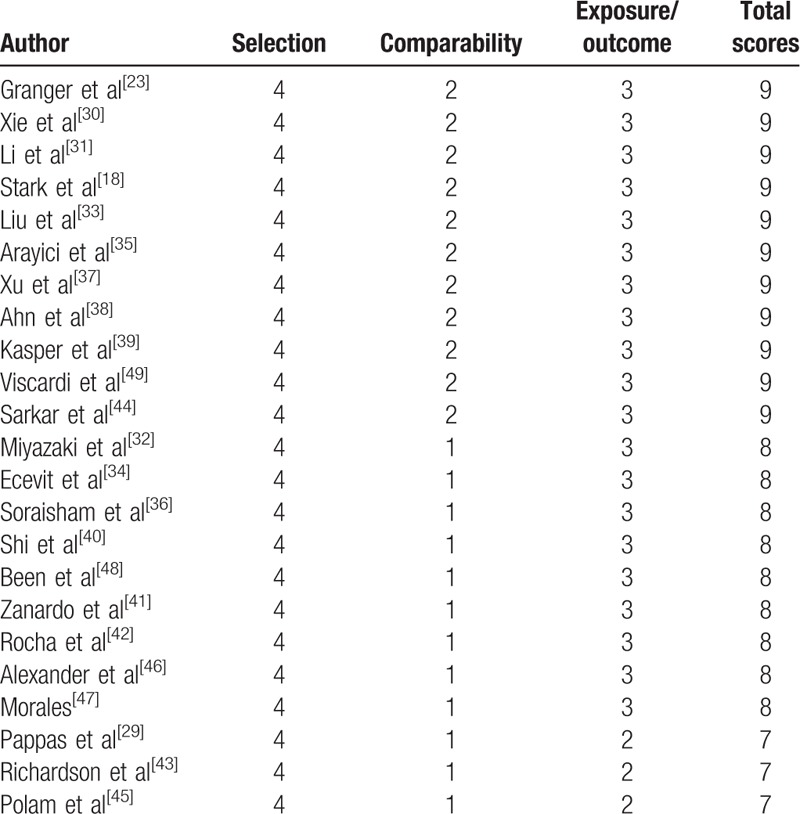
Newcastle–Ottawa Quality Assessment Scale results for the included studies.

### 3.3 Antenatal infection and IVH

**Figure 2:**
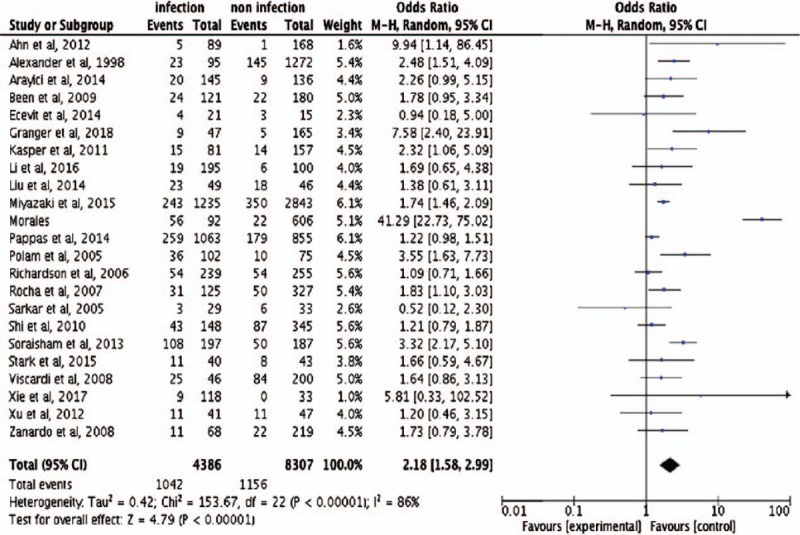
Forest plots of antenatal infection and intraventricular hemorrhage (IVH). Odds ratio >1 indicates that compared with noninfection, antenatal infection could increase the risk of IVH in preterm infant.

**Figure 3:**
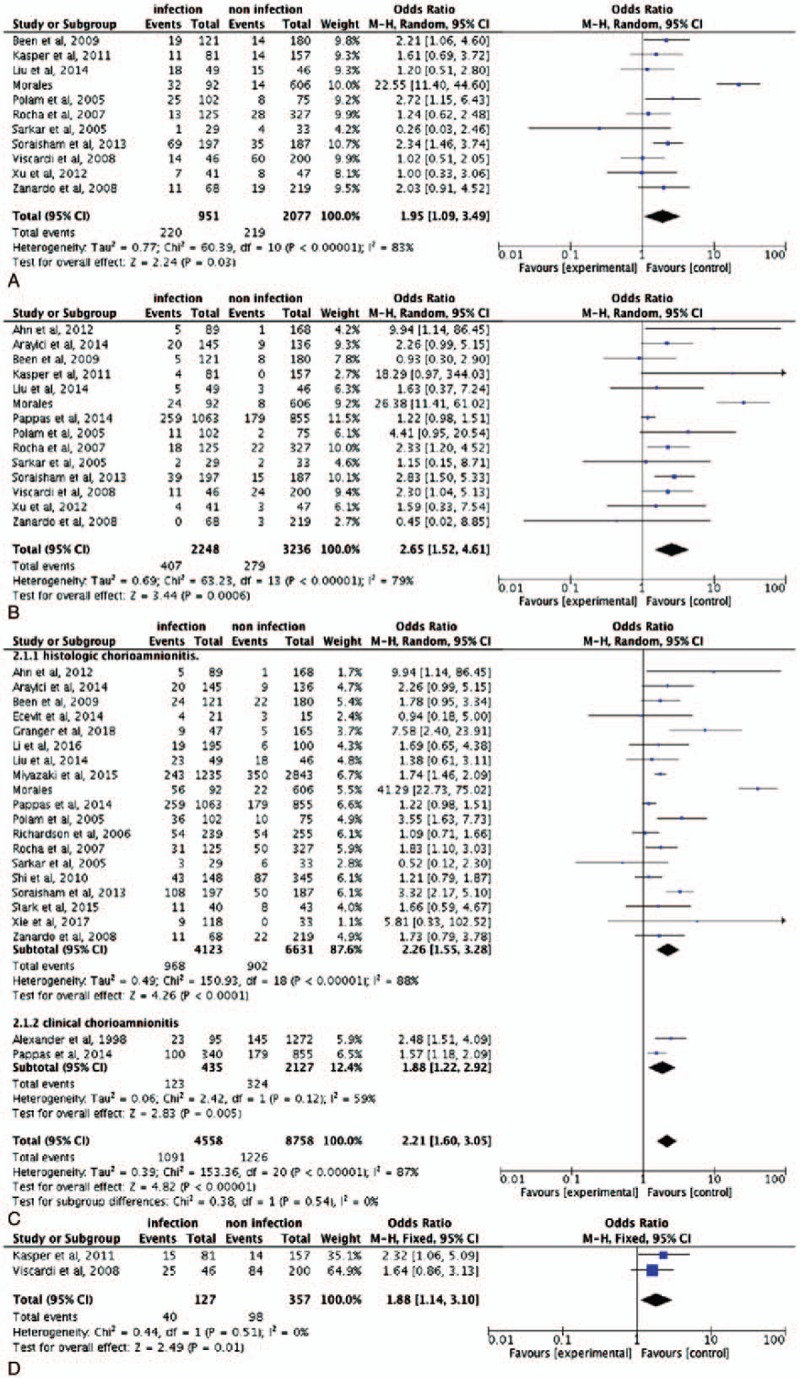
Forest plots of antenatal infection and intraventricular hemorrhage (IVH). (A) Forest plots of antenatal infection and mild IVH. (B) Forest plots of antenatal infection and severe IVH. (C) Forest plots of chorioamnionitis and IVH. (D) Forest plots of ureaplasma and IVH. Odds ratio >1 indicates that compared with noninfection, antenatal infection could increase the risk of IVH in preterm infant.

Evaluating all 23 of the studies, the overall effect sizes for IVH were significantly different (OR 2.18, 95% CI 1.58–2.99) (23 trials/12693 infants) between those with and without antenatal infection, indicating that antenatal infections increase the risk of IVH in preterm infants (Fig. 2). Additionally, antenatal infection increased not only the risk of mild IVH (OR 1.95, 95% CI 1.09–3.49) (11 trials/3028 infants), but also severe IVH (OR 2.65, 95% CI 1.52–4.61) (14 trials/5484 infants) in premature infants (Fig. 3A and B).

To determine whether the type of infection is associated with IVH, we conducted subgroup analyses of different types of antenatal infection. Seventeen of the studies assessed the impact of histologic chorioamnionitis and found it increases the risk of IVH (OR 2.26 95% CI 1.55–3.28) (19 trials/10754 infants). In addition, IVH (OR 1.88, 95% CI 1.22–2.92) (2 trials/2562 infants) was statistically significantly increased in the babies whose mothers had clinical chorioamnionitis. The OR and 95% CI was 1.88 (1.14–3.10) (2 trials/484 infants) for ureaplasma (Fig. 3C and D). The fixed-effects model was selected for subgroup of ureaplasma because of the *I*^2^ = 0% and *P* = .51. For the other group, the random-effects model was selected.

### 3.4 Sensitivity analysis

After removing all of the low-quality studies, there were still significant changes in the risk of IVH (OR 1.92, 95% CI 1.35–2.75) (11 trials/2008 infants), severe IVH (OR 2.33, 95% CI 1.45–3.74) (7 trials/1267 infants) and histologic chorioamnionitis group (OR 2.14, 95% CI 1.24–3.70) (9 trials/1430 infants). However, there were no longer statistically significant differences in the mild IVH group (OR 1.13, 95% CI 0.75–1.70) (5 trials/729 infants). Notably, the result of ureaplasma group was not affected because the 2 included studies were of high-quality. There was no result for clinical chorioamnionitis because both studies were of low quality.

When we only included articles without statistical differences in baseline GA between infection and no infection group, there was a significantly increased risk for severe IVH (OR 3.04, 95% CI 1.02–9.05) (7 trials/3380 infants), in clinical chorioamnionitis (OR 1.57, 95% CI 1.18–2.09) (1 trial/1195 infants) and ureaplasma group (OR 2.32, 95% CI 1.06–5.09) (1 trial/238 infants). However, no significant differences was obsereved for IVH (OR 2.19, 95% CI 0.81–5.95) (8 trials/3463 infants), mild IVH (OR 1.85, 95% CI 0.40–8.48) (5 trials/1181 infants), and in histologic chorioamnionitis group (OR 2.36, 95% CI 0.91–6.17) (9 trials/3731 infants).

### 3.5 Publication bias

**Figure 4:**
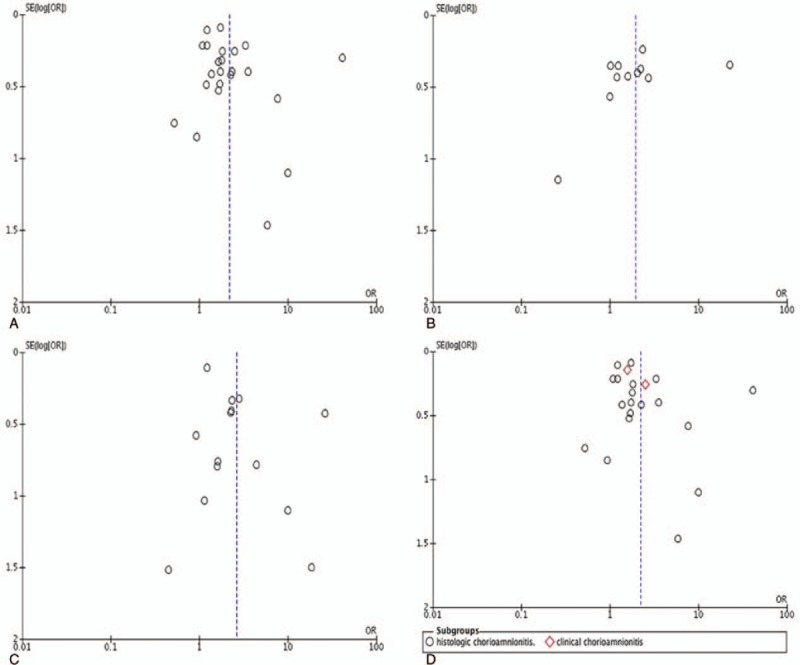
(A) Funnel plot of antenatal infection and intraventricular hemorrhage (IVH). (B) Funnel plot of antenatal infection and mild IVH. (C) Funnel plot of antenatal infection and severe IVH. (D) Funnel plot of chorioamnionitis and IVH.

The publication bias was first evaluated visually by the funnel plot (Fig. 4). Then, we performed Egger test to explore potential publication bias. For the subgroups of clinical chorioamnionitis and ureaplasma, Egger test could not be performed because of the low number of studies. For the other group, the results showed that the publication bias were not significant (*P* > .05).

### 3.6 GRADE assessment

**Figure 5:**
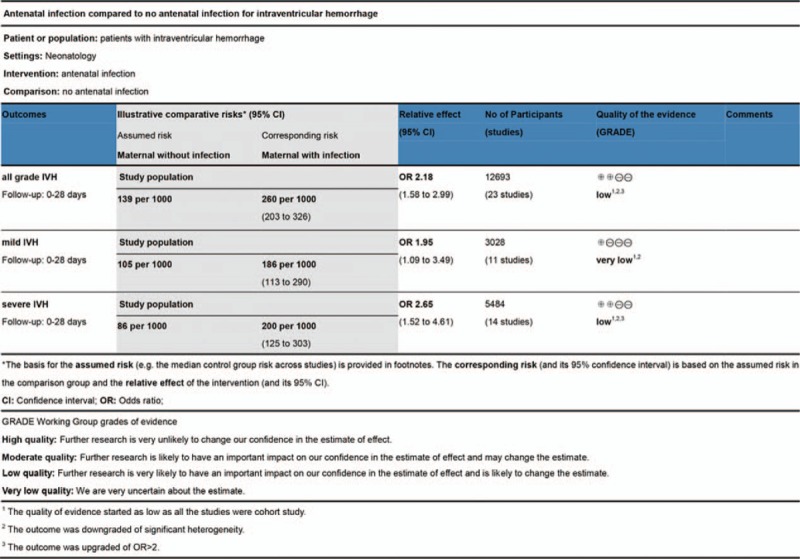
Quality evaluation by GRADE tool for antenatal infection versus no infection.

The qualities of the evidence were low for IVH (all grade) and severe IVH, and very low for mild IVH (Fig. 5). The quality started as low as all studies were cohort studies and that outcomes were downgraded because of significant heterogeneity and upgraded by OR >2.

## 4 Discussion

Our meta-analysis verified the profound relationship between antenatal infection and IVH in preterm infants from current evidence. Our findings extend the understanding of previous reports. The results from 23 cohort studies with 13605 infants indicated that antenatal infection increased the incidence of IVH in preterm infants (OR 2.18, 95% CI 1.58–2.99). The risk of both mild (OR 1.95, 95% CI 1.09–3.49) and severe IVH (OR 2.65, 95% CI 1.52–4.61) was increased by antenatal infection, compared with no infection.

More than 50% of preterm fetuses delivered before 30 weeks’ gestation have chorioamnionitis, rather than presentations of sepsis/pneumonia syndromes.^[50]^ In our review, the most frequently reported antenatal infection was chorioamnionitis. Antenatal infection including histologic chorioamnionitis (OR 2.26 95% CI 1.55–3.28) and clinical chorioamnionitis (OR 1.88, 95% CI 1.22–2.92) contributes to the development of IVH. As the pathogenesis of IVH is not completely known, our finding that antenatal infection increases the risk of development of IVH highlights a new perspective for the etiology of IVH. This may be of benefit to the prevention of this common preterm complication by reducing antennal infection. The reported impact of antenatal infections on IVH among preterm infants adds up to the well-known maternal–infant interaction.

### 4.1 Overall completeness and applicability of evidence

We have attempted to identify all available published and unpublished data for the relationship of antenatal infection and IVH in preterm birth. The included studies were performed in neonatal intensive care units in Australia, China, Japan, United States, Canada, Netherlands, Italy, and Portugal. Data yielded across the globe may be widely representative. Thus, the evidence of our review is applicable to most hospital settings in mid- or high-income countries. New evidence from low-income country would support the overall applicability of the data. The average GA of neonates in included studies was preterm infants <33 weeks with birth weight <1900 g. Thus, these findings should be cautiously applied to late preterm (34–36 weeks) infants. The included studies were published from 1987 to 2018. Although there is a large time span, the majority of studies were published in the era of 2000s, and the diagnostic criteria for IVH have remained constant, which makes the results applicable for current practice.

### 4.2 Advantages and limitations

Our meta-analysis has several advantages. First, this is the first systematic review to summarize the current evidence regarding the relationship between antenatal infection and risk of IVH. The severity of IVH (all grades IVH, mild IVH, and severe IVH) and the type of antenatal infection (histologic chorioamnionitis, clinical chorioamnionitis, and ureaplasma) were carefully assessed. The relationship between antenatal infection and IVH was generally supported by the statistically significantly effects from results of our meta-analysis. This indicates that antenatal infection may lead to increased risk of IVH in preterm infants. Second, meta-analyses of observational studies are prone to biases and confounding factors owing to intrinsic nature of the original studies. We minimized the bias by restricting our analyses to cohort studies, and excluding traditional case–control studies, which are prone to recall and interviewer bias. Third, sensitivity analysis provides robust evidence for the association in this review. Results were generally consistent when we applied sensitivity analysis. Most of the sensitivity analysis results have not changed significantly after discarding low-quality studies. Fouth, the results of funnel plot and Egger test showed no significant publication bias, which means the results have low risk of selection bias. Finally, our meta-analysis included studies from different countries and the preterm infants included in the studies ranged from extremely low borth weight to LBW, indicating that our findings are broadly representative.

Our study has some limitations. First of all, all of the included studies were observational studies, which may be influenced by selection bias. The quality of the evidence was graded as “low or very low,” because of entirely of observational studies design and high heterogeneity. However, the relationship between antenatal infection and IVH cannot be investigated in RCTs for ethical or methodological reasons. Observational research is useful for assessing etiology and is the only choice for this topic to provide evidence for clinical decision. In addition, we restricted our search to English and Chinese databases. Research published in other language was not included. This may lead to selection bias from language.

One important issue is the complicated relationship among infection, prematurity, and IVH. It is well known that a lower GA is associated with a higher frequency of IVH. Besides, chorioamnionitis is much more frequent in low GA. In this review, some included studies had a significant GA difference between the infection and noninfection group. To assess possible impact of antenatal infection on IVH through GA, we performed sensitivity analysis by eliminating studies with significant GA difference. We found there was a significant increased risk for severe IVH, in the clinical chorioamnionitis and ureaplasma group. Increased trends were obsereved for IVH, mild IVH, and in the histologic chorioamnionitis group, although there were no statistically significant differences. These outcomes may indicate that antenatal infection leads to IVH not only based on lower GA.

### 4.3 Implications for practice and research

It has been reported that routine use of an antenatal infection screen and treat program could decrease the risk of preterm birth.^[3]^ Given the evidence between antenatal infection and IVH, researchers should carefully consider the need of antenatal infection screen and treat program for IVH in the future researches, which may prevent the preterm infant from avoidable IVH.

## 5 Conclusion

In conclusion, we found that antenatal infection may play an important role in predisposing preterm newborns to IVH and we stress the importance of antenatal infection prevention. Our meta-analysis was limited by the low or very low quality of evidence of GRADE assessment, indicating that additional well-designed studies should be performed to explore the role of antenatal infections in IVH.

## Author contributions

**Data curation:** Jinglan Huang, Junjie Meng, tao xiong.

**Formal analysis:** Jinglan Huang, Junjie Meng.

**Funding acquisition:** tao xiong, Yi Qu, Dezhi Mu.

**Investigation:** Jinglan Huang, Junjie Meng, tao xiong, Yibin Wang, Huiqing Wang.

**Methodology:** Jinglan Huang, Junjie Meng.

**Project administration:** Yi Qu.

**Resources:** tao xiong.

**Software:** Jinglan Huang, Junjie Meng, tao xiong.

**Supervision:** Imti Choonara, tao xiong, Yibin Wang, Huiqing Wang, Yi Qu, Dezhi Mu.

**Validation:** Imti Choonara, Yibin Wang, Huiqing Wang, Dezhi Mu.

**Visualization:** Imti Choonara, tao xiong, Yibin Wang, Huiqing Wang, Yi Qu, Dezhi Mu.

**Writing – original draft:** Jinglan Huang, Junjie Meng, Yibin Wang, Huiqing Wang, Yi Qu.

**Writing – review & editing:** Imti Choonara, tao xiong, Dezhi Mu.
